# A Novel Copper-Binding Peptide That Self-Assembles Into a Transparent Antibacterial and Antiviral Coating

**DOI:** 10.3389/fbioe.2021.736679

**Published:** 2021-10-20

**Authors:** Daniel Boas, Meital Reches

**Affiliations:** The Institute of Chemistry and The Center for Nanoscience and Nanotechnology, The Hebrew University of Jerusalem, Jerusalem, Israel

**Keywords:** metal-binding peptide, antiviral coating, antibacterial coating, transparent coating, nanomaterials

## Abstract

The health, economy, and quality of life all over the world are greatly affected by bacterial infections and viral outbreaks. Bacterial cells and viruses, such as influenza, can spread through contaminated surfaces and fomites. Therefore, a possible way to fight these pathogens is to utilize antibacterial and antiviral coatings, which reduce their numbers on contaminated surfaces. Here, we present a novel short peptide that can self-assemble, adhere to various surfaces, and bind different metal ions such as copper, which provides the surface with antibacterial and antiviral properties. For these functions, the peptide incorporates the amino acid 3,4-dihydroxyphenylalanine (DOPA), which provides the peptide with adhesive capabilities; a diphenylalanine motif that induces the self-assembly of the peptide; the metal-binding hexahistidine sequence. Our results demonstrate that the coating, which releases monovalent cuprous ions and hydrogen peroxide, provides the surfaces with significant antibacterial and antiviral properties. Additionally, the coating remains transparent, which is favorable for many objects and especially for display screens.

## 1 Introduction

The worldwide outbreak of the coronavirus disease 2019 (COVID-19), caused by the novel severe acute respiratory syndrome coronavirus 2 (SARS-CoV-2), has drastically affected the health, economy, and quality of life of many countries across the world (Acter et al., 2020). This is the third emergence in humans of a highly lethal coronavirus in the last 18 years, after the 2002–2003 SARS pandemic and the ongoing 2012 Middle East respiratory syndrome (MERS) outbreak (de Wit et al., 2016). In addition to the novel coronavirus, influenza viruses can also cause respiratory infections. These viruses are the source of four pandemics since the 20th century, including the devastating 1918 Spanish flu, and the continuous seasonal outbreaks in the annual period known as the flu season (Saunders-Hastings and Krewski, 2016). Although the major transmission pathway of COVID-19 appears to be aerosols (Harvey et al., 2021), this disease, influenza, and other viral diseases that affect the respiratory tract can spread through contaminated surfaces and fomites (Vasickova et al., 2010; Gholipour et al., 2020; Sun and Ostrikov, 2020; Elbadawy et al., 2021).

The importance of fighting nosocomial infections, which are mainly caused by bacterial contamination of surfaces, is reinforced as the resistance of these pathogenic bacteria to antibiotics expands (Cloutier et al., 2015). Recent data shows that over 44% of the most frequently reported pathogens across healthcare-associated infections are caused specifically by gram-negative bacteria (Weiner-Lastinger et al., 2020). Immunocompromised patients in hospitals are especially at risk from nosocomial infections. However, bacterial contamination of surfaces is not a problem present solely in hospitals and medical equipment, rather it can affect any place or object where transmission of these bacteria to humans is possible, such as in public transportation, faucets, elevator buttons, door handles, keyboards, and touch screens.

The burdens of both the viral outbreaks and the bacterial infections could be mitigated *via* a single coating with the dual functionality of being antiviral and antibacterial. Different materials were used as a coating that combines antiviral and antibacterial functions, such as silver nanoparticles, TiO_2_, small-molecule organics, polymers, and Cu-containing surfaces (Kim et al., 2015; Si et al., 2018; Gurunathan et al., 2020; Balasubramaniam et al., 2021). The antiviral and antimicrobial mechanism of these materials involves different pathways, such as reactive oxygen species (ROS) production, contact killing, and membrane depolarization by the dissolution of metal ions. Antibiotic resistance usually occurs because the antibiotic targets a single biochemical process (Balasubramaniam et al., 2021). Therefore, using a material that simultaneously targets different processes could be advantageous in avoiding this phenomenon. While antiviral and antibacterial coatings have been previously reported, there is a need for a coating that can be easily applied to various surfaces, self-assemble to a stable coating, and utilize many of the antibacterial and antiviral capabilities of Cu, while maintaining the original appearance of the coated material.

Here, we present a novel peptide that self-assembles into a coating with both antiviral and antibacterial activities. The peptide, NH_2_-DOPA-(Phe)_2_-(His)_6_-OH, utilizes three elements to achieve three distinct functions: 1) The amino acid 3,4-dihydroxy-L-phenylalanine (DOPA) that is abundant in the mussel adhesive proteins (Waite and Tanzer, 1981; Lee et al., 2007). This amino acid can adhere strongly to various organic and inorganic surfaces using several different interactions, such as hydrogen bonding, hydrophobic interactions, and metal-coordination interactions (Amini et al.; Lee et al., 2006; Kan et al., 2020). The addition of DOPA to a peptide can provide it with adsorption capabilities (Maity et al., 2014; Yuran et al., 2018). 2) The diphenylalanine motif—this element is in the core of the Alzheimer’s ß-amyloid polypeptide and is involved in the formation of amyloid fibrils (Reches and Gazit, 2003). The diphenylalanine motif was shown to induce a self-assembly process via π-stacking. Adding a self-assembling motif to the peptide can be beneficial as such peptides can be biocompatible, easy to synthesize and to apply, they can exhibit diverse structures and functions, and have considerable potential for the design of nanomaterials or biomaterials (Aggeli et al., 2001; Hartgerink et al., 2001; Zhang, 2003; Reches and Gazit, 2006; Hamley, 2007; Ulijn and Smith, 2008; Cui et al., 2010; Yan et al., 2010; Ikezoe et al., 2012; Wang et al., 2019). In addition, hydrophobic moieties around DOPA were shown to enhance its adhesive capabilities and strengthen the interactions between adhesive proteins (Wei et al., 2013). Adding this segment to the peptide can aid its self-assembly process and presumably enhance its coacervation via stronger intermolecular interactions between the peptide molecules (Maity et al., 2014; Yuran et al., 2018). 3) A sequence of six histidine residues. This sequence is commonly used for protein isolation via affinity chromatography because of its strong interaction with metal ions, e.g., Ni(II), Co(II), or Cu(II) (Bornhorst and Falke, 2000; Blankespoor et al., 2005). For example, the binding of N-acetyl hexahistidine to Cu(II)-nitrilotriacetic acid (NTA) and to Ni(II)-NTA were shown to have dissociation constants of 27.1 and 69.4 µM, respectively (Mehlenbacher et al., 2015).

The combination of these three elements results in a peptide that can self-assemble, adhere to various surfaces, and form coordination bonds with different metal ions. Since Cu displays an antimicrobial and antiviral activity through diverse pathways, we chose it for this study (Kim et al., 2015; Balasubramaniam et al., 2021). This peptide can be used to coat various surfaces and provide them with antibacterial and antiviral activity, while the appearance of the surfaces remains unaffected, which is advantageous for many potential fomites.

## 2 Materials and Methods

### 2.1 Materials

Fmoc-DOPA(acetonide)-OH, Fmoc-Phe-OH, and Fmoc-His(Trt)-OH were purchased from GL Biochem (Shanghai, China). Triisopropylsilane (TIPS) was purchased from TCI Europe N.V. (Zwijndrecht, Belgium). 1-[Bis(dimethylamino)methylene]-1H-1,2,3-triazolo[4,5-b]pyridinium 3-oxide hexafluorophosphate (HATU) was purchased from Matrix Innovation, Inc. (Saint-Hubert, QC, Canada). Dimethylformamide (DMF), dichloromethane (DCM), piperidine, diethyl ether, N,N-Diisopropylethylamine (DIPEA), acetonitrile, and tri-fluoroacetic acid (TFA) were purchased from Bio-Lab (Jerusalem, Israel). Sodium phosphate monobasic monohydrate was purchased from Mallinckrodt Chemicals (Chesterfield, United Kingdom). Difco Nutrient Agar and Difco LB broth, Lennox were purchased from BD Biosciences (San Jose, CA, United States). HCL fuming (37%), tryptic soy broth (TSB), lecithin, tween 80, 2,2′-azino-bis(3-ethylbenzothiazoline-6-sulfonic acid) (ABTS) diammonium salt, peroxidase from horseradish (HRP), and hydrogen peroxide solution (30 wt. %) were purchased from Merck (Darmstadt, Germany). Lysogeny broth (LB) was purchased from Becton Dickinson (BD) (Franklin Lakes, NJ, United States). Agarose was purchased from Lifegene (Mevo Horon, Israel). Sodium dodecyl sulfate (SDS) was purchased from J.T. Baker Inc. (Phillipsburg, NJ, United States). Pierce BCA protein assay kit was purchased from Thermo Fisher Scientific (Waltham, MA, United States). NaCl was purchased from Fisher Scientific (Hampton, NH, United States). D_2_O was purchased from Cambridge Isotope Laboratories, Inc. (Tewksbury, MA, United States). NaBH_4_, CuCl_2_, and CuCl were purchased from Acros Organics (Fair Lawn, NJ, United States). 2-Chlorotrityl chloride resin (1.0–1.6 mmol/g, 100—200 mesh) was purchased from Chem-Impex International, Inc. (Wood Dale, IL, United States). *Escherichia coli* (Migula) Castellani and Chalmers strain B (ATCC 11303) and FDA strain Seattle 1946 (ATCC 25922) and *E. coli* bacteriophage T4 (ATCC 113030-B4) were obtained from American Type Culture Collection (Manassas, VA, United States).

### 2.2 Substrates

Ti substrates: Si wafers with a diameter of 2 inches were coated with 50 nm Ti (as measured by a quartz crystal microbalance) using electron beam evaporation (TFDS-141E, VST) at a rate of 2 Å/sec. Si substrates: Si wafers with a diameter of 10 cm were used. Mica surfaces: mica discs with a 9.9 mm diameter (Ted Pella Inc., Redding, CA, United States) were used. Glass substrates: glass microscope slides (76 mm × 26 mm × 1 mm, Paul Marienfeld GmbH & CO. KG, Lauda-Königshofen, Germany) were diced into 1 × 1 cm^2^ pieces before use (7100 2 in. Pro-Vectus, ADT).

### 2.3 Peptide Synthesis

The peptides were synthesized manually by Fmoc solid-phase peptide synthesis (SPPS). 2-Chlorotrityl chloride resin was used (0.25 mmol scale). The amino acids were used with a 5-fold excess, and they were activated using a DIPEA/HATU mixture (4 equiv. and 3.9 equiv., respectively) for 4 min. The coupling procedure was carried for 1 h and a Kaiser test was performed after each coupling. The Fmoc protecting group was removed by stirring with a solution of 20% piperidine in DMF for 20 min. The washing procedure included washing twice with DMF, methanol, DCM, and DMF again. Before the cleavage of the peptide, the resin was washed three times with DMF and DCM. The cleavage reaction was performed using a mixture of TFA/TIPS/TDW (10 ml, 95:2.5:2.5) for 3 h. The cleaved solution was evaporated via nitrogen bubbling, precipitated with diethyl ether, and centrifuged. The crude peptide product was dissolved in an ACN/TDW mixture (5 ml, 1:1) and lyophilized.

### 2.4 Peptide Characterization

#### 2.4.1 High-Performance Liquid Chromatography Analysis

Analytical reverse-phase HPLC analysis was performed using a Waters Alliance HPLC with UV detection (at 220 and 254 nm) and an XSelect C18 column (3.5 µm 130 Å, 4.6 mm × 150 mm). The peptides were eluted using a linear gradient of ACN (with 0.1% TFA) in TDW (with 0.1% TFA), at a flow rate of 1 ml/min, 30°C.

#### 2.4.2 Liquid Chromatography Mass Spectrometry (LC/MS) Analysis

LC(UV)MS/MS analysis was performed using Agilent 6520 Q-TOF analyzer (Agilent Technologies, Santa Clara, CA, United States).

#### 2.4.3 Quartz Crystal Microbalance With Dissipation (QCM-D) Analysis

The peptides’ adsorption and the metal-binding capabilities were studied using QCM-D (Q-sense, Biolin Scientific). The measurements were performed in a flow module E1 system with SiO_2_ sensors with a fundamental resonant frequency of 5 MHz (Qsense). The sensors were cleaned according to the supplier’s instructions. The experiments were performed under flow-through conditions using a digital peristaltic pump (IsmaTec Peristaltic Pump, IDEX). The peptide (1.5 mg/ml), control peptide (1.5 mg/ml), and CuCl_2_ (0.8 mg/ml) were dissolved in a filtered Tris buffer solution (pH = 8.5, 10 mM). The solutions were injected circularly into the sensor crystal chamber at a rate of 0.1 ml/min. The peptide solution flowed for ∼4 h, the control peptide for ∼80 min, and the CuCl_2_ solution for ∼2 h. Between and after the coatings, the sensor was washed with the same Tris buffer solution. The adsorbed mass was calculated according to the Sauerbrey equation using the 5th overtones. The displayed data is representative.

### 2.5 Surface Modification

Mica disks were cleaved before each use. 1 × 1 cm^2^ Ti or Si surfaces were sonicated for 15 min in ethanol, washed with TDW, and dried with nitrogen. 1 × 1 cm^2^ SiO_2_ surfaces were cleaned with Oxygen/Plasma (Atto, Diener Electronic) for 10 min, immersed in SDS (2%) for 30 min, washed with TDW and dried under N_2_. Immediately before coating with the peptide, the surfaces were treated by O_2_ plasma for 1 min (Atto, Diener Electronic, Ebhausen, Germany). The surfaces were immersed in the peptide solution (1.5 mg/ml, 1.1 mM, in a filtered Tris buffer at pH = 8.5, 10 mM, with an ionic strength of 154 mM with NaCl) for 4 h, at room temperature. The coated surfaces were washed three times with 1 ml of the same Tris buffer solution and dried with nitrogen. After that, the surfaces were immersed in the CuCl_2_ solution (0.8 mg/ml, 4.6 mM, in the same Tris buffer solution) for 2 h, at room temperature. The washing step was repeated. Lastly, the surfaces were immersed in an ice-cold NaBH_4_ solution (0.3 mg/ml, 9.1 mM in the same Tris buffer) for 1 h, at room temperature, and the washing step was repeated.

### 2.6 Coating Characterization

#### 2.6.1 High-Resolution Scanning Electron Microscopy and Energy-Dispersive X-Ray Spectroscopy (EDS) Analyses

Si and mica surfaces were cleaned and coated in the same methods mentioned above. The mica surface was coated with Ir using Quorum Q150V S Plus Sputter Coater. sputter coater. HR-SEM images were taken using Sirion XL30 SFEG (Thermo Fisher, former FEI), operating at 5 kV, and equipped with XMAX SDD EDS detector (Oxford Instruments, Abingdon, United Kingdom) on an Inca Energy 450 platform. Extra-high-resolution SEM (XHR-SEM) images were acquired using Magellan 400L (Thermo Fisher, former FEI), operating at 2 kV.

#### 2.6.2 Atomic Force Microscopy

Mica surfaces were cleaned as mentioned above and one surface was coated using the three-step procedure. AFM images were taken in NanoWizard 3 instrument (JPK, Berlin, Germany) at AC mode with a silicon tip that has a spring constant of 6 N/m (Aspire, Team Nanotec GmbH, Villingen-Schwenningen, Germany).

#### 2.6.3 X-Ray Photoelectron Spectroscopy Analysis

XPS measurements were taken using Kratos AXIS Supra spectrometer (Kratos Analytical Ltd., Manchester, United Kingdom). The spectra were acquired using the Al-K_α_ monochromatic radiation X-ray source (1,486.6 eV). The sample takeoff angle was 90° (normal to the analyzer) and the vacuum pressure in the analyzing chamber was maintained at 2 × 10^–9^ Torr. High-resolution XPS spectra were collected for C 1s, N 1s, Ti 2p, O 1s, and Cu 2p with pass energy 20 and 0.1 eV step size. The binding energies were calibrated using C 1s peak energy as 285.0 eV. The data were analyzed using ESCApe processing program (Kratos Analytical Ltd.) and Casa XPS (Casa Software Ltd.). The thickness of a coated Si surface was calculated based on the formula:

$$d=\lambda_{0}\sin\theta \ln\left(\frac{N_{s}\lambda_{s}I_{0}}{N_{0}\lambda_{0}I_{s}}+1\right)$$ (1)

where *d* is the thickness in nm, *I*_*0*_ and *I*_*s*_ are the intensities of the peaks from the layer and the substrate, respectively, the substrate is the 2p signal from Si and the layer is the sum of the intensities of C 1s, O 1s, and N 1s peaks, *θ* is the takeoff angle (here sin⁡θ=1 ), and *N*_*0*_ and *N*_*s*_ are the volume densities. The inelastic mean free path parameters for the layer *λ*_*0*_ and the substrate *λ*_*s*_ were assumed as 3.3 and 3.09 nm, respectively (calculated using QUASES-IMFP-TPP2M software) (Taylor, 2007).

#### 2.6.4 Attenuated Total Reflectance Fourier-Transform Infrared Spectroscopy Analysis

The spectra of a bare Ti surface, as well as p-, PC-, and PCN-coated Ti surfaces was measured using a Nicolet 6700 FTIR spectrometer (Thermo Fisher Scientific, Waltham, MA, United States) with Ge-ATR arrangement (VariGATR, Harrick Scientific, Pleasantville, NY, United States). The experimental setup for all spectra included 3,000 scans, an applied force of 350 N, an incident angle of 65°, and 4 cm^−1^ resolution.

#### 2.6.5 Fourier-Transform Infrared Spectroscopy Analysis

A peptide solution (1.5 mg/ml), a 1:1 mixture of the peptide solution and a CuCl_2_ solution (0.8 mg/ml), and a 1:1:1 mixture of the peptide, CuCl_2_, and NaBH_4_ (0.3 mg/ml) solutions were deposited on CaF_2_ plates and dried under vacuum. The peptide and mixtures on the plates were resuspended in D_2_O and dried under vacuum twice to remove the H_2_O hydration layer from the plate. The infrared spectra were recorded using a Nicolet 6700 FTIR spectrometer with a deuterated triglycine sulfate (DTGS) detector (Thermo Fisher Scientific, Waltham, MA, United States). The experimental setup included 2000 scans and 4 cm^−1^ resolution. The absorbance peaks were determined using the OMNIC analysis software (Nicolet).

#### 2.6.6 Contact Angle Measurements

Contact angle measurements were taken using a Theta Lite optical tensiometer (Attention, Finland). Each result is composed of an average of 3 repeats with 20 measurements on each surface.

#### 2.6.7 Transmittance Measurements

The absorption spectra of a clean SiO_2_ surface and a SiO_2_ surface coated with the peptide, CuCl_2_, and treated with NaBH_4_ were measured using a UV/Vis spectrophotometer (Shimadzu, UV-1650PC, Kyoto, Japan). The transmittance (*%T*) of each wavelength was calculated from the absorbance (A) according to the relation: $\%T=10^{\left(2-A\right)}$ .

#### 2.6.8 Plaque Assay

The assay was based on ISO 21702 standard with several changes. Three clean SiO_2_ surfaces and three SiO_2_ surfaces coated with the peptide, CuCl_2_, and treated with NaBH_4_ were used in each of the three experiments. For the control experiments, three surfaces coated with just the peptide, the peptide and CuCl_2_, and the peptide with NaBH_4_ treatment were used. A single colony of *E. coli* 11303 was transferred to 10 ml nutrient broth (2% LB broth) and incubated overnight at 37°C, 120 rpm. From a heated nutrient agar solution (0.5% NaCl), 2 ml was transferred to each of the wells in 6-wells plates and left to solidify. *E. coli* bacteriophage T4 stock solution was diluted to a concentration of 10^6^ plaque-forming units (PFU)/ml in LB phage (0.8% LB broth, 0.5% NaCl). From the bacteriophage solution, 16 µl were dropped on each sample and a 0.8 × 0.8 cm^2^ parafilm was placed on top to evenly spread the bacteriophages on the surface. The samples were incubated at room temperature for 24 h. The phages were removed from the surfaces after 24 h by washing 3 times with SCDLP broth (2 ml). The samples were shaken in the SCDLP broth (3% TSB, 0.1% lecithin, and 0.7% tween 80) for 15 min at 150 rpm. A 10-fold dilution was prepared from each sample to a dilution of 10^–1^. Warm agarose (0.6%, 1 ml), the *E. coli* starter (25 µl), and the appropriately washed bacteriophage solutions (20 µl) were added into test tubes. Each tube was gently shaken, and its contents poured over the previously prepared agar plates. The plates were left at room temperature for a few minutes until the agarose solidified and placed at 37°C in an incubator overnight. The plates were then removed from the incubator, the plaques from each well were counted, and the PFUs were determined according to the dilution.

#### 2.6.9 Antibacterial Assay

The assay was based on JIS Z 2801 standard with several changes. Three clean SiO_2_ surfaces and three SiO_2_ surfaces coated with the peptide, CuCl_2_, and treated with NaBH_4_ were used in each of the three experiments. A single colony of *E. coli* 25922 was transferred to TSB solution (20 ml) and incubated overnight at 37°C, 120 rpm. The solution was centrifuged at 4,000 rpm for 10 min, the supernatant was removed, and the precipitate resuspended in PBS buffer (10 mM, pH = 7.0, 154 mM NaCl, 1% TSB, 10 ml). This stage was repeated for three more times. The optical density (OD) of the solution was measured at 600 nm using a UV/Vis spectrophotometer (Shimadzu, UV-1650PC, Kyoto, Japan) and the bacteria concentration was diluted accordingly to 10^6^ colony forming units (CFU)/ml. A 20 µl drop from the bacteria solution was deposited in the middle of each surface and covered with a 0.8 × 0.8 cm^2^ parafilm layer. The surfaces were incubated for 24 h in a humid atmosphere at 37°C. Afterward, the surfaces were placed in test tubes with PBS buffer (1 ml). Each test tube was separately sonicated for 1 min and vortexed for 15 s, then 10^–1^ to 10^–3^ dilutions were prepared using PBS buffer, and 20 µl stripes were pipetted on agar plates (0.5% NaCl). The plates were incubated overnight at 37°C, the colonies were counted and the CFUs were determined according to the dilution.

#### 2.6.10 Bicinchoninic Acid Assay

A CuCl solution (0.1 mg/ml) was prepared by first dissolving the CuCl in HCl (37%) and diluting the solution in TDW. This solution was then diluted to a total of 8 different CuCl concentrations. A drop of TDW (100 µl) was placed on a SiO_2_ surface that was coated with the peptide, CuCl_2_, and treated with NaBH_4_. In addition, several months old surfaces were used, with and without repeating the coating steps with CuCl_2_ and NaBH_4_. The surfaces were left for different time periods in a humid environment. The Pierce BCA protein assay kit reagents A and B were combined at a 50:1 ratio and 200 µl were added from this mixture to 25 µl from the 8 CuCl dilutions and to 25 µl from the water deposited on the coated surfaces. The mixtures were gently mixed, incubated at 37°C for 30 min, cooled down to room temperature and their absorbance was measured at 562 nm using a UV/Vis spectrophotometer (Shimadzu, UV-1650PC, Kyoto, Japan). A calibration curve was made using the CuCl dilutions and the amount of Cu(I) released from the coated surfaces to the water was calculated.

#### 2.6.11 ABTS Assay

An H_2_O_2_ solution (1 mM) was prepared in PBS buffer (100 mM, pH = 7.0). This solution was then diluted to a total of 8 different H_2_O_2_ concentrations. A drop of PBS (100 µl) was placed on SiO_2_ surfaces, coated with the peptide, CuCl_2_, and treated with NaBH_4_. In addition, several months old surfaces were used, with and without repeating the coating steps with CuCl_2_ and NaBH_4_. The surfaces were left for different time periods in a humid environment. A 2,2′-azino-bis(3-ethylbenzothiazoline-6-sulfonic acid) (ABTS) solution (8.6 mM) and an HRP solution (5 mg/ml) were prepared in PBS. The ABTS solution (4 µl) was mixed with the 8 H_2_O_2_ dilutions and with the PBS deposited on the coated surfaces (40 µl). The HRP solution was added (2 µl), the mixtures were vortexed for 30 s, and their absorbance was measured at 734 nm using a UV/Vis spectrophotometer (Shimadzu, UV-1650PC, Kyoto, Japan). A calibration curve was made using the H_2_O_2_ dilutions and the amount of H_2_O_2_ released from the surfaces to the PBS was calculated.

### 2.7 Statistics

The data from the plaque and antibacterial assays represent three independent experiments. In each plaque assay experiment, three uncoated and three coated surfaces were used. Each plaque assay was performed in duplicates and each antibacterial assay was performed in triplicates. The results for the control plaque assay, as well as the assays meant to display the effect of NaCl, were produced from one experiment. All the graphs were plotted using Origin software (OriginLab Corporation). The plaque assay and antibacterial test results of the PCN-coated surfaces were compared using Student’s two-sample *t*-test assuming unequal variances. The plaque assay results for the control surfaces were compared using one-way ANOVA test with Tukey-Kramer post hoc analysis. Statistically significant results were marked with one asterisk for *p* < 0.01 (*) and two asterisks for *p* < 0.001 (**).

## 3 Results and Discussion

### 3.1 Peptide Design

**FIGURE 1:**
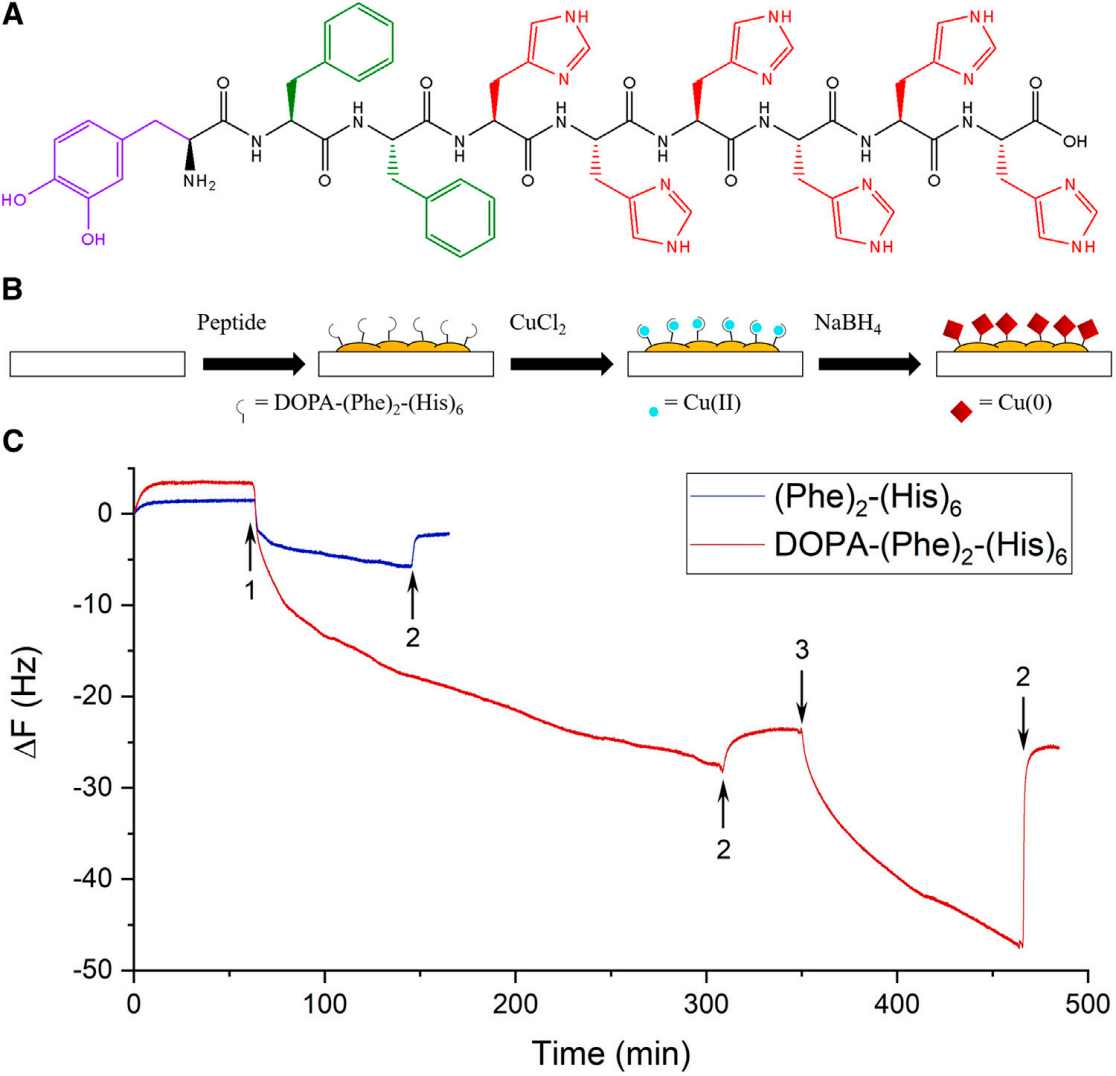
Formation of a peptide coating. **(A)** The molecular structure of the peptide. The purple color depicts the amino acid DOPA, green Phe, and red His. **(B)** A scheme representing the three coating steps. **(C)** Representative real-time QCM-D measurements of the peptide (red) and a control peptide without DOPA (blue). The 5^th^ overtones are presented. The arrows indicate the addition of the peptides (1), washing with tris (2), and the addition of a CuCl_2_ solution (3).

We designed the trifunctional peptide NH_2_-DOPA-(Phe)_2_-(His)_6_-OH. This design incorporates three elements: 1) the amino acid DOPA, which provides the peptide with adsorption capabilities to most substrates (Lee et al., 2007); 2) a diphenylalanine segment, which facilitates the peptide’s self-assembly process through π stacking, can aid the adhesion of the DOPA, and stabilize the coacervation of the peptide (Reches and Gazit, 2003); and 3) a hexahistidine part, which can generate coordinate bonds with various metal ions, e.g., Ni^2+^ and Cu^2+^ (Figure 1A) (Liu et al., 2003; Blankespoor et al., 2005). We suggest that the employment of a single peptide sequence with the three functions mentioned can be beneficial in designing an antiviral and antibacterial coating.

### 3.2 Surface Modification

The surface modification was performed using a three-step procedure in which the surfaces were immersed in a peptide solution (P), a Cu solution (PC), and an NaBH_4_ solution (PCN) to reduce the copper (Figure 1B) (Banerjee et al., 2003). These steps were meant to allow the formation of a peptide layer on the surface, form bonds between the histidine residues of the peptide and Cu ions, and finally, reduce these ions to cover the peptide layer with Cu crystals. Additionally, since DOPA can also bind metal ions, separating the adhesion of the peptide on the surface via DOPA from the addition of Cu is important to avoid competition between DOPA and hexahistidine. The three steps were performed in a Tris buffer solution with an ionic strength of 154 mM with NaCl. After every immersion, the surface was rinsed three times with 1 ml of the buffer solution, to ensure the removal of weakly attached residues and to preliminarily demonstrate the stability of the coating.

The oxidation of DOPA is considered to assist its adhesion by allowing multiple interactions with the surface. To promote this process, the procedure was performed at alkaline conditions using Tris buffer (Lee et al., 2006; Xi et al., 2009; Das and Reches, 2016; Mirshafian et al., 2016; Yuran et al., 2018). Additionally, these conditions were favored because at lower pH values the imidazole groups on the histidine residues are protonated and can no longer bind metal ions (Moyer et al., 2014; Zheng et al., 2016).

The interactions of the salt ions with water could abate the interactions between water and the polar amino acids, i.e., histidine and DOPA. This could encourage the coacervation of the peptide *via π* stacking interactions between the phenylalanine residues, thus forming a peptide layer more easily in a solution with higher ionic strength (Wei et al., 2014). The effect of the salt on the coacervation of the peptide can be seen in the solutions of the peptide, Cu(II), and their mixture, with and without NaCl (Supplementary Figure S2). While no turbidity can be seen in the solutions without NaCl, the coacervation is clearly visible in the peptide-Cu mixture with NaCl. Additionally, NaCl is used in His-tag purification as it can reduce weak ionic interactions and prevent nonspecific binding, which could be formed in this case between the peptide and the surface or other components in the solution.

The adhesion of the peptide and its ability to bind Cu ions were investigated by QCM-D analysis. The peptide flowed for ∼4 h and caused a substantial change in frequency, which indicated the adsorption of the peptide to the SiO_2_ sensor (Figure 1C). After washing the sensor with the buffer solution, only a small change in frequency was observed, which points to the stability of the peptide coating. The changes in dissipation were ∼1 × 10^–6^, which suggests the formation of a rigid film (Supplementary Figure S3) and supports the use of the Sauerbrey equation to resolve the adsorbed mass on the sensor (Bordes et al., 2010). The loaded mass/area for the peptide was ∼96 ng/cm^2^. Afterward, a Cu(II) solution was introduced and caused a large change in frequency, which decreased after washing with the buffer. However, a smaller change remained, corresponding to a loaded mass/area value of ∼9 ng/cm^2^. This value is expected given that the Cu ions bound to the peptide have a lower molar mass and it corresponds to a ratio of ∼2 Cu ions per each peptide molecule, as is expected from previous studies (Watly et al., 2014).

To demonstrate the importance of DOPA for the peptide’s adhesion, we synthesized the peptide NH_2_-(Phe)_2_-(His)_6_-OH (without DOPA) and investigated its adhesion to the surface using QCM-D. This peptide caused a small frequency change, which was further reduced by washing with the buffer. The loaded mass/area for this peptide was ∼12 ng/cm^2^, an eighth of the value for the DOPA-containing peptide, which indicates the significant effect of DOPA on the adhesion.

### 3.3 Surface Characterization

**FIGURE 2:**
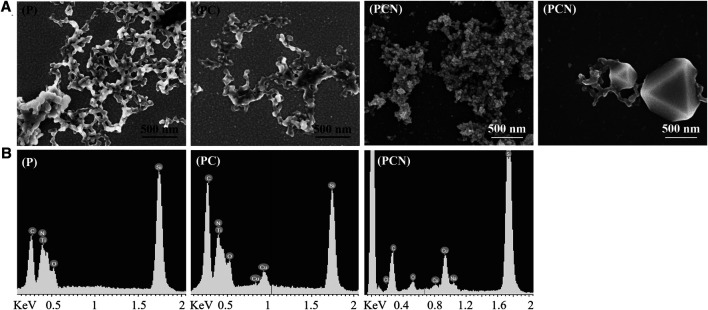
Characterization of the P-, PC-, and PCN-coated surfaces. **(A)** SEM images of the peptide network-like nanostructures on P- and PC-coated surfaces. The same structures appear on a PCN-coated surface as well as the appearance, on the same surface, of CuNPs embedded on the peptide nanostructures. **(B)** EDS analysis of P-, PC-, and PCN-coated surfaces.

Surfaces coated with the peptide (P), peptide and Cu (PC), and those coated with the peptide and Cu and treated with NaBH_4_ (PCN) were analyzed using SEM. The SEM images show a network-like nanostructure of the peptide (Figure 2A). Similar structures were previously received from a hexahistidine peptide’s assembly with Zn ions (Huang et al., 2019). The structure does not differ greatly with the addition of Cu ions but appears rougher after the reduction treatment with NaBH_4_. Additionally, structures that appear to be Cu nanoparticles (CuNPs) embedded in the peptide network-like nanostructures were visible on some areas of the PCN-coated surface. EDS analysis showed the presence of the peptide on all three surfaces and of Cu bound to the peptide on the PC-and PCN-coated surfaces (Figure 2B).

To view the differences between a clean surface to areas that are not covered with the peptide nanostructures on a PCN-coated surface, freshly cleaved and PCN-coated mica surfaces were imaged using AFM. The topography of the bare mica was unobservable due to the low signal-to-noise ratio, while that of the PCN-coated surface was distinct (Supplementary Figure S4). The difference can be seen also from the different roughness parameters (Rq) measured for the bare mica surface (0.3 nm) and for the PCN-coated surface (1.3 nm). This indicates that there is a presence of a thin peptide layer on areas that appeared in the SEM analysis to be uncovered.

**FIGURE 3:**
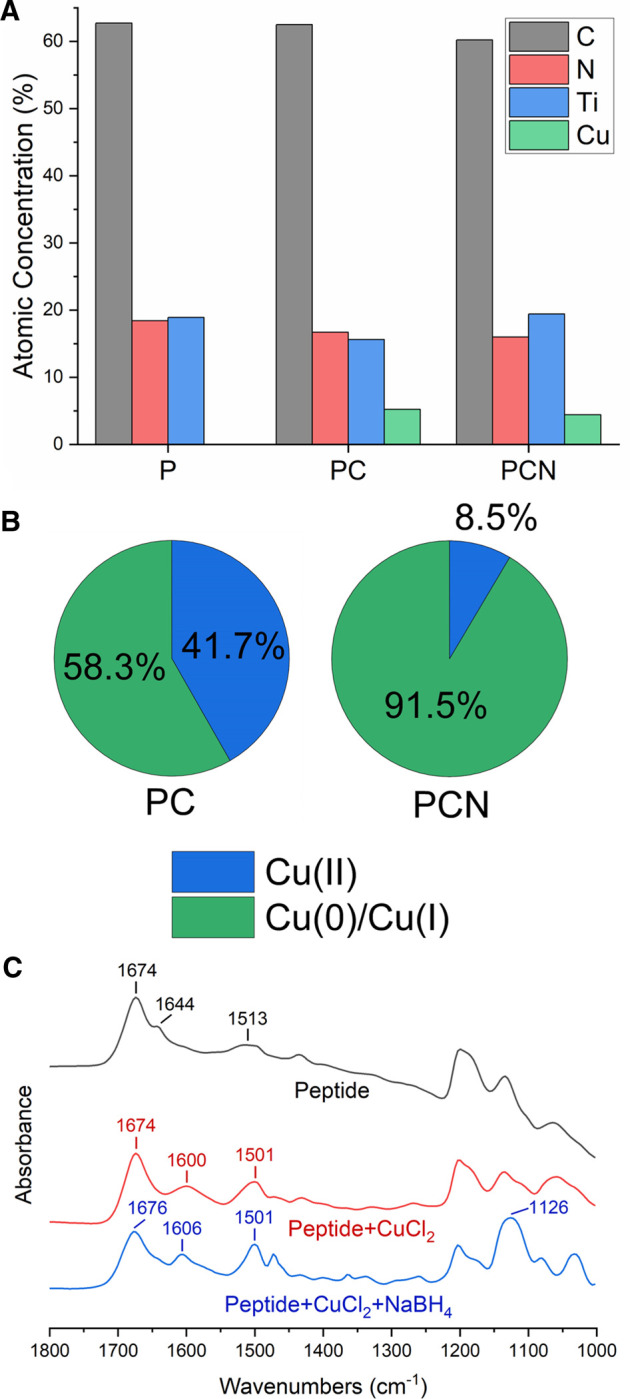
Composition and structural analysis of the P-, PC-, and PCN-coated surfaces. **(A)** Atomic concentrations (%) of carbon (black), nitrogen (red), titanium (blue), and copper (green) on P-, PC-, and PCN-coated surfaces. **(B)** Area (%) below the Cu(II) related peaks (blue) and Cu(0) or Cu(I) related peaks (green) in the Cu 2p spectra for PC and PCN-coated surfaces. **(C)** FTIR spectra of the peptide (black), a mixture of the peptide and CuCl_2_ (red), and a mixture of the peptide, CuCl_2_, and NaBH_4_ (blue).

XPS was used to further examine P-, PC-, and PCN-coated surfaces. The results supported the presence of the peptide and of the Cu on the surfaces (Figure 3A). The reduction step with NaBH_4_ did not appear to remove the peptide layer. Additionally, the resulting Cu 2p spectra indicated that the reduction step was successful as it increased the area below the Cu(0) and Cu(I) related peaks from 58 to 92% at the expense of the area below the Cu(II) related peaks (Figure 3B). This also suggested that there is some level of Cu(II) reduction done by the peptide itself, before introducing the reducing agent. In addition, the XPS measurements allow calculating the average thickness of the PCN coating, which was established at 6.5 nm (Razvag et al., 2013).

To obtain additional information on the interactions present in the coating, we performed FT-IR analyses. We measured CaF_2_ plates coated with the peptide, a mixture of the peptide and CuCl_2_, and a mixture of the peptide, CuCl_2_, and NaBH_4_. The broad peak at 1,600 cm^−1^ and the narrower one at 1,606 cm^−1^ for the PC-and PCN-coated surfaces respectively, could be attributed to the degree of crystallinity of the structures on the plate (Huang et al., 2019). This could be explained due to the formation of Cu crystals on the PC-and PCN-coated plates and is supported by the XPS results, which show a majority of reduced Cu atoms also on the PC-coated surface (Figure 3B). The peak at 1,513 cm^−1^ in the spectrum of the P-coated plate could be indicative of imidazole ring stretching (Figure 3C). The shift and narrowing of this peak at the spectra of the PC-and PCN-coated plates could point to interactions between the imidazole ring of the histidine residues and the Cu. Additionally, the peak at 1,126 cm^−1^ in the spectrum of the PCN-coated plate could be associated with the stretching vibration of the CN group in the imidazole (Nivedhini Iswarya et al., 2017). These data support the existence of the peptide’s interactions with Cu ions or with metallic Cu through the histidine residues.

**FIGURE 4:**
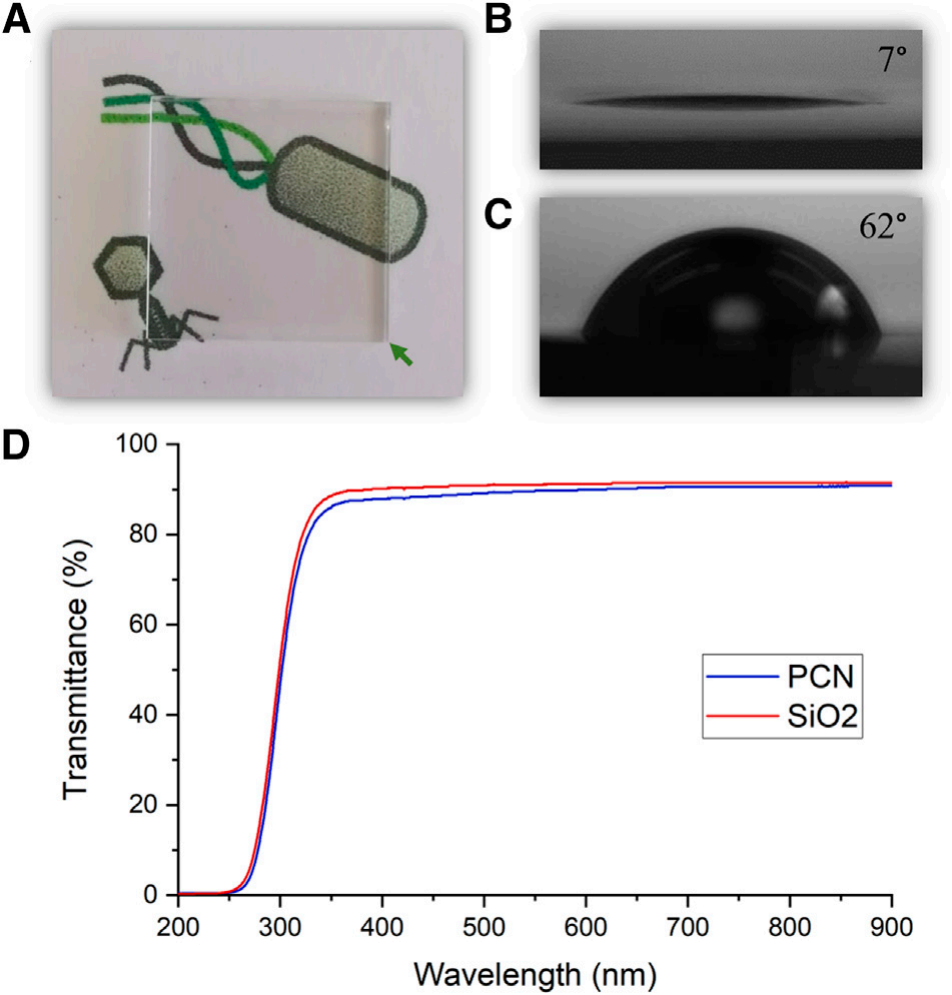
Transparency of the PCN-coated SiO_2_ surface. **(A)** A photo of a PCN-coated surface indicating the coating’s transparency. **(B,C)** Photos depicting the contact angles of a clean SiO_2_ surface **(B)** and a PCN-coated SiO_2_ surface **(C)**. **(D)** Visible light range transmittance spectra of a clean SiO_2_ surface and a PCN-coated surface.

The coating procedure resulted in an optically transparent coating, as expected from the coating’s thickness (Figure 4A). The difference between a clean surface and the coated surface, while not visible to the naked eye, can be revealed in contact angle measurements. A clean glass surface presented a contact angle of 7° and the coated surface showed an increase of the contact angle to 62° (Figures 4B,C). Transmittance spectra of a clean glass surface and a PCN-coated surface were measured and confirmed the transparency of the coating in the visible light range (Figure 4C). A transparent coating could be advantageous for covering touch screens, phone cases and screen protectors, office partitions and dividers, face shields, windows, etc.

### 3.4 Antiviral Activity

**FIGURE 5:**
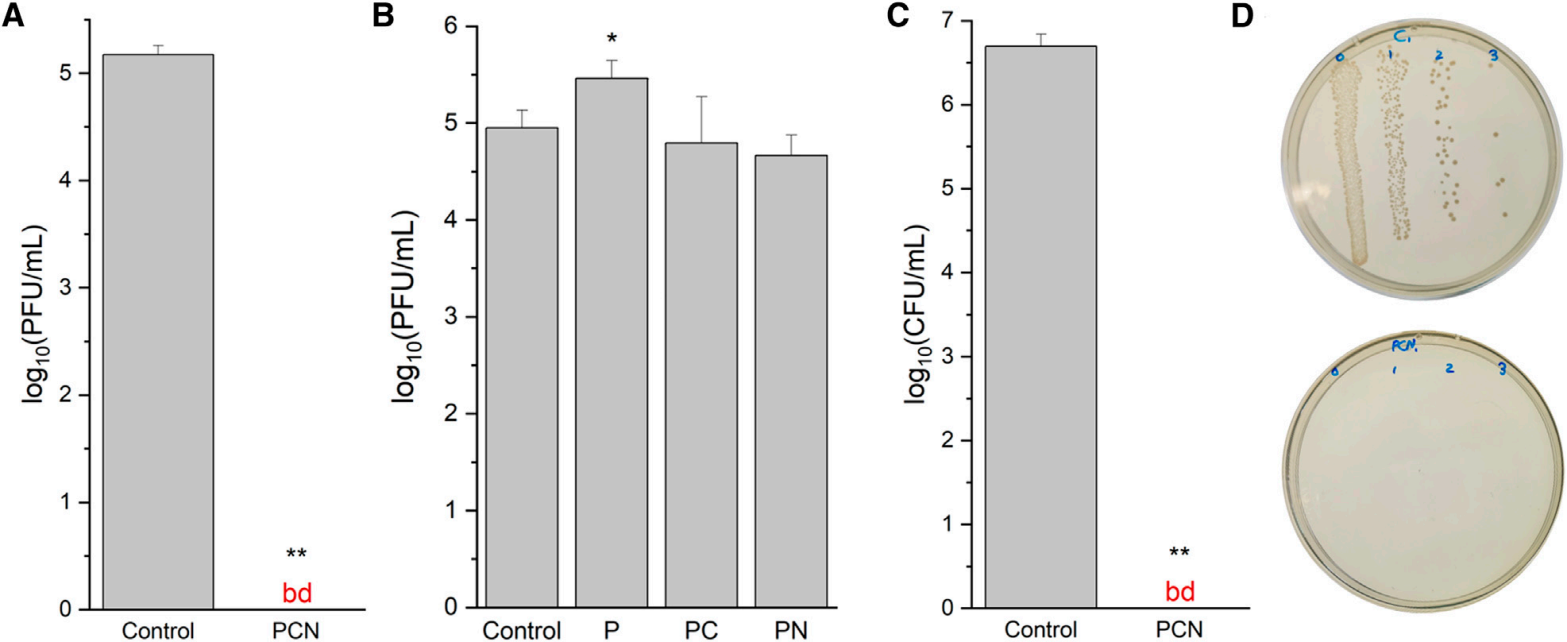
Antiviral and antibacterial activity of the peptide-coated surfaces. **(A)** Virucidal activity of a PCN-coated SiO_2_ surface against a clean SiO_2_ surface (control). The data are representative of three biological repeats, each performed as duplicates. **(B)** Virucidal activity of P-, PC-, and PN-coated SiO_2_ surfaces against a clean SiO_2_ surface. **(C)** Bactericidal activity of a PCN-coated SiO_2_ surface against a clean SiO_2_ surface. The data are representative of three biological repeats, each performed as triplicates. **(D)** A photo of an agar plate from the antibacterial assay with bacteria from a clean SiO_2_ surface (top) and with bacteria from a PCN-coated surface (bottom). bd—below detection.

To assess the antiviral activity of the PCN-coated surface we performed plaque assays using T4 bacteriophages (ATCC 113030-B4) and *E. coli* (ATCC 11303) as their host. The number of bacteriophage plaques after incubation on a coated surface, when compared to those that were cast on uncoated surfaces, can provide insight to the coating’s antiviral and virucidal capabilities. As presented in Figure 5A, the coating reduced the number of phages by more than 5 orders of magnitude (100%) when compared to uncoated SiO_2_ surfaces. The phages that are incubated on the surfaces are washed and then placed on agar plates. This means that if the surface was simply preventing the adhesion of the phages, they would still be detected as plaques. Thus, these assays reveal that the PCN-coated surfaces are virucidal as they kill the phages.

To identify the necessary factors in the coating for the antiviral activity, P-and PC-coated SiO_2_ surfaces, as well as surfaces coated with the peptide and treated with NaBH_4_ (PN) were also examined with bacteriophage plaque assays. From these assays, presented in Figure 5B, it appears that the peptide coating by itself could promote the survivability of the phages on the surface. This may be explained due to the emergence of niches in the peptide’s network structure, in which the phages can shelter from the exposed SiO_2_ surface. On the other hand, PC-and PN-coated surfaces did not display an explicit reduction of the bacteriophage numbers. The results for these surfaces were not consistent, resulting in relatively large standard deviation values, which strengthens their inferiority to the PCN-coated surface.

The importance of the salt to the formation of a coating with strong antiviral activity can be inferred from the plaque assays of PCN-coated surfaces with and without NaCl in the buffer solutions used in the surface modification steps. Without NaCl, the number of phages was reduced by 39% and with NaCl the number was reduced by 80% (Supplementary Figure S7). These assays were performed on Ti surfaces, with a 3 h incubation time of the surfaces with the phages instead of 24 h.

### 3.5 Antibacterial Activity

The PCN-coated surface was tested for bactericidal activity on *E. coli* (ATCC 25922). The number of bacterial colonies on the coated surface was reduced by more than 6 orders of magnitude (100%) when compared to a clean SiO_2_ surface (Figure 5C). As with the bacteriophage plaque assays, these antibacterial assays also reveal the bactericidal activity of the PCN-coated surfaces. These results, when combined with the virucidal activity, reveal the effectiveness of the coating’s antiviral and antibacterial capabilities.

### 3.6 Suggested Antiviral and Antibacterial Mechanism

Different Cu materials display a pleiotropic effect as they involve several antiviral and antimicrobial pathways, negating the development of antibiotic resistance. The exact antiviral and antimicrobial mechanism of Cu remains uncertain, but several processes have been proposed (Balasubramaniam et al., 2021). Cu ions were shown to bind bacterial cells, causing membrane depolarization that leads to leaks and ruptures. Another process associated with Cu ions, which is also apparent in CuNPs, involves the production of ROS that can damage the genetic material and the lipid membranes. Specifically, Cu(II) ions have a lesser effect on viruses while the more potent Cu(I) ions were shown to possess significant antiviral abilities (Sunada et al., 2012; Kim et al., 2015). The antiviral and antimicrobial abilities of metallic Cu via contact killing were proposed by Grass et al. to involve four processes: 1) Cu dissolution from the surface causes cell damage, 2) the cellular membrane loses its integrity and its contents, 3) the Cu ions induce ROS formation, and 4) the genetic material undergoes degradation (Grass et al., 2011).

**FIGURE 6:**
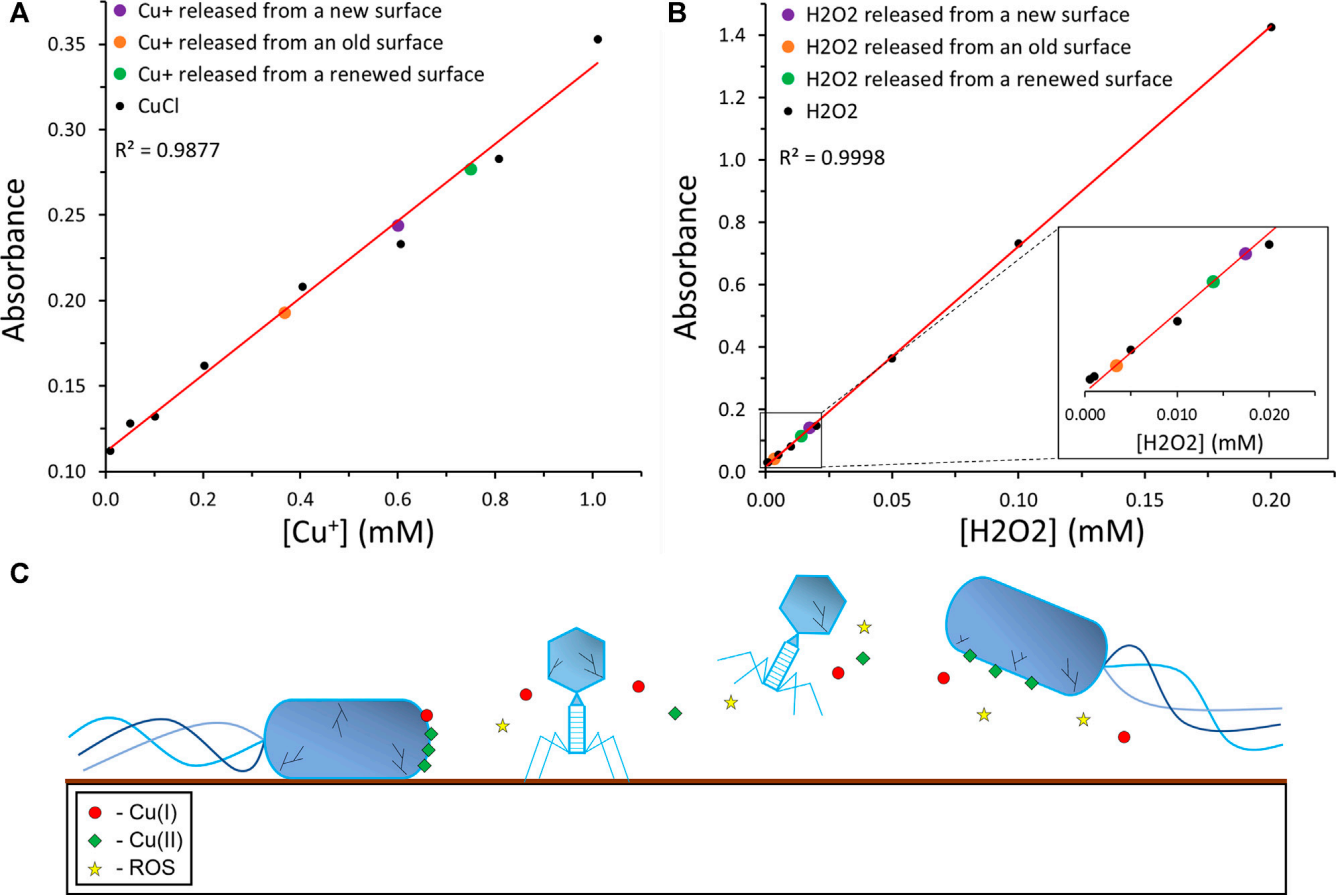
**|** Proposed antiviral and antibacterial mechanism of the coating. **(A)** A calibration curve of Cu(I) ions was performed using a BCA assay. The known Cu(I) concentration data points are depicted in black, their linear trendline in red, and the data point corresponding to the Cu(I) concentration released from the new, old, and renewed PCN-coated surfaces are depicted in purple, orange, and green, respectively. **(B)** A calibration curve of H_2_O_2_ was performed using an ABTS assay. The known H_2_O_2_ concentration data points are depicted in black, their linear trendline in red, and the data point corresponding to the H_2_O_2_ concentration released from the new, old, and renewed PCN-coated surfaces are depicted in purple, orange, and green, respectively. **(C)** The suggested mechanism of the antibacterial and antiviral activity of the PCN-coated surface.

To clarify the origin of the coating’s antiviral and antibacterial activity, we performed an assay that will reveal the release of Cu(I) ions released from the surface. The assay was carried out with BCA that forms a complex with Cu(I) ions, which retains a prominent linear absorption at 562 nm. A calibration curve was made using CuCl solutions with known concentrations and the amount of Cu(I) ions released from the PCN-coated surface after different time periods was determined (Figure 6A; Supplementary Figure S8A). The ratio between the Cu^+^ ions released from the new surface after 24 h, to the number of phages or bacteria placed on the surfaces in the plaque assays and antibacterial assays is ∼2 × 10^12^.

In addition, we performed an assay with ABTS, to reveal the production and release of H_2_O_2_ from the surface. ABTS^2-^ can be oxidized by the enzyme horseradish peroxidase (HRP) in the presence of hydrogen peroxide to ABTS^•−^, which absorbs at 734 nm (Nir et al., 2019). A calibration curve was made using H_2_O_2_ solutions with known concentrations and the amount of H_2_O_2_ released from the PCN-coated surfaces after different time periods was determined (Figure 6B; Supplementary Figure S8B). While the amount of H_2_O_2_ released may not seem high enough to kill the phages or the bacteria in a solution, one should note that the H_2_O_2_ is released from the surface onto the bacteria or phages that contact the surface. Furthermore, the ratios between the H_2_O_2_ molecules released from the new surface after 24 h, to the number of phages or bacteria placed on the surfaces in the plaque assays and antibacterial assays are ∼7 × 10^10^ and ∼5 × 10^10^, respectively.

Absolute values for the release of Cu(I) ions and H_2_O_2_ should not be concluded from these assays, as the variation between different PCN-coated surfaces is possible. However, the trend of the release of Cu(I) and H_2_O_2_ is clear. In addition, the possibility of renewing the activity of an old PCN-coated surface is indicated by these assays. A freshly prepared surface released ∼60 nmol Cu(I) and ∼1.7 nmol H_2_O_2_ after 24 h. After several months, these values decreased to ∼37 and ∼0.3 nmol, respectively. Yet, after repeating the coating steps with the copper and NaBH_4_, the old surfaces restored their Cu(I) and H_2_O_2_ release to ∼75 and 1.4 nmol, respectively. The possibility of reactivating old PCN-coated surfaces without repeating the peptide coating simplifies the real-life employment of this coating and its effectiveness.

Since both Cu(I) and H_2_O_2_ have well-studied antibacterial and antiviral properties, these results could provide insight to the mechanism of those properties in the PCN coating (Figure 6C) (Sunada et al., 2012; Kim et al., 2015). The inclusion of the potent antiviral and antibacterial abilities of Cu(I) ions and H_2_O_2_ with the possible dissolution of the ROS-generating, bacteria-binding Cu(II) ions, and the properties of metallic Cu and CuNPs, could result in a strong antiviral and antibacterial coating that kills both viruses and bacteria. Additionally, the release of Cu(I) and H_2_O_2_ from the old PCN-coated surface, as well as the possibility of renewing these surfaces, strengthen the potential of this coating.

## 4 Conclusion

We presented the design of an optically transparent metal-binding peptide coating with antiviral and antibacterial activity. Analysis of the peptide demonstrated its adhesive capabilities, ability to bind Cu(II) ions and to form nanostructures on the surface. While the experiments were mostly performed on SiO_2_ surfaces, since DOPA and DOPA-containing peptides are known to bind various surfaces (Maity et al., 2014; Yuran et al., 2018), we suggest that this peptide can also be used to coat various surfaces and objects. The coating procedure is simple as the coating forms spontaneously and does not require special equipment. This suggests that a coating kit for everyday uses may be a feasible idea. The coating displayed significant antiviral and antibacterial activity in bacteriophage plaque assays and antibacterial assays when the peptide nanostructures were covered with a Cu crystal layer. While possessing notable antiviral and antimicrobial activity, the coating is not visible to the naked eye as it has high transparency in the visible light range. This could be highly advantageous as the coating could be used for covering surfaces such as glass and screens. The combination of metallic Cu, CuNPs, Cu(I) and Cu(II) ions, and H_2_O_2_, can illuminate the result of an efficient antiviral and antibacterial coating.

Such a coating could prove useful for covering various surfaces with an antiviral and antibacterial material without altering their appearances. Surfaces of masks and visors, gloves, medical appliances, door handles, keyboards, and touch screens could be proper candidates for this coating. Such a coating could also prove to possess antifouling and antifungal abilities, as is expected from the properties of Cu materials. Since most COVID-19 infections appear to be transmitted through aerosols, applying this coating on air filters could be advantageous. Additionally, the possibility of using this peptide for purposes of drug delivery combined with the pH-dependent release potential of hexahistidine is intriguing (Huang et al., 2019; Zhang et al., 2020). At times when there are wide-spread epidemics or pandemics caused by viruses, such as influenza virus or coronavirus, an antiviral and antimicrobial coating like the one presented here could provide valuable means to combat the spread of the disease, prevent bacterial infections of immunodeficient patients in hospitals, and to recover faster from the state of emergency.

## Data Availability

The original contributions presented in the study are included in the article/Supplementary Material, further inquiries can be directed to the corresponding author.
