# Supplementary material for: A Novel Copper-Binding Peptide That Self-Assembles Into a Transparent Antibacterial and Antiviral Coating
Source: Front Bioeng Biotechnol. 2021 Oct 20;9:736679. doi: 10.3389/fbioe.2021.736679 (PMC8564293; doi:10.3389/fbioe.2021.736679)
Supplement: Supplementary file 1 [file DataSheet1.docx]

A Novel Copper-Binding Peptide That Self-Assembles into a Transparent Antibacterial and Antiviral Coating

Daniel Boas^1^, Meital Reches^1*^

^1^The Institute of Chemistry and The Center for Nanoscience and Nanotechnology, The Hebrew University of Jerusalem, Edmond J. Safra Campus, Givat Ram, Jerusalem, Israel

*** Correspondence:**Meital Reches
meital.reches@mail.huji.ac.il

Keywords: metal-binding peptide, antiviral coating, antibacterial coating, transparent coating, nanomaterials

### Fourier-Transform Infrared (FTIR) Spectroscopy Analysis

To decipher the secondary structure of the peptide on the surface, ATR-FTIR spectroscopy measurements of a clean Ti surface and P-, PC-, and PCN-coated Ti surfaces were performed. As a result of the low signal of the peptide, no secondary structure could be inferred from the spectra. This could be explained by the nanometric thickness of the coating. However, the formation of a coating can be derived from the disappearance of the bare Ti peak at 876 cm^-1^ on the coated surfaces (**Supplementary Figure 6**) (Maity et al., 2014). Because of the lower signal in ATR-FTIR, we performed FTIR analyses that have a higher signal-to-noise ratio (Bindig et al., 2003). We measured CaF_2_ plates coated with the peptide, a mixture of the peptide and CuCl_2_, and a mixture of the peptide, CuCl_2_, and NaBH_4_. The strong peaks at 1674 cm^-1^ for the P- and PC-coated plates and at 1676 cm^-1^ for the PCN-coated plate indicate that the peptide formed β-turns, which were unaffected by the addition of CuCl_2_ and NaBH_4_ (**Figure 3C**) (Barth, 2007; Miller et al., 2013). A lower peak at 1644 in the P-coated plate could be associated with a disordered structure of the peptide or random coil structures (Haris and Chapman, 1995). These structures appear to diminish when Cu(II) ions are added, suggesting that the ions can strengthen the interactions present in the peptide structures, causing the β-turn structures to be predominant.


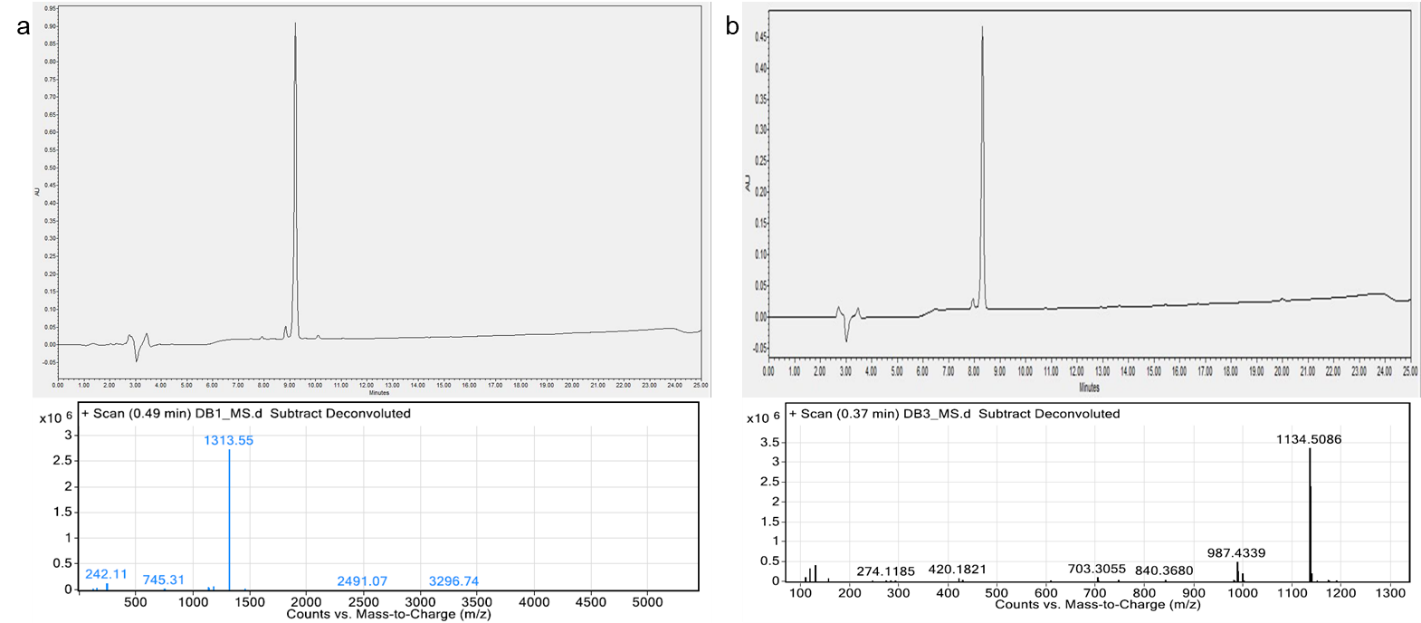


**FIGURE 1 |** (**A**) Analytical HPLC chromatogram and MS analysis of the peptide. (**B**) Analytical HPLC chromatogram and MS analysis of the peptide without DOPA.


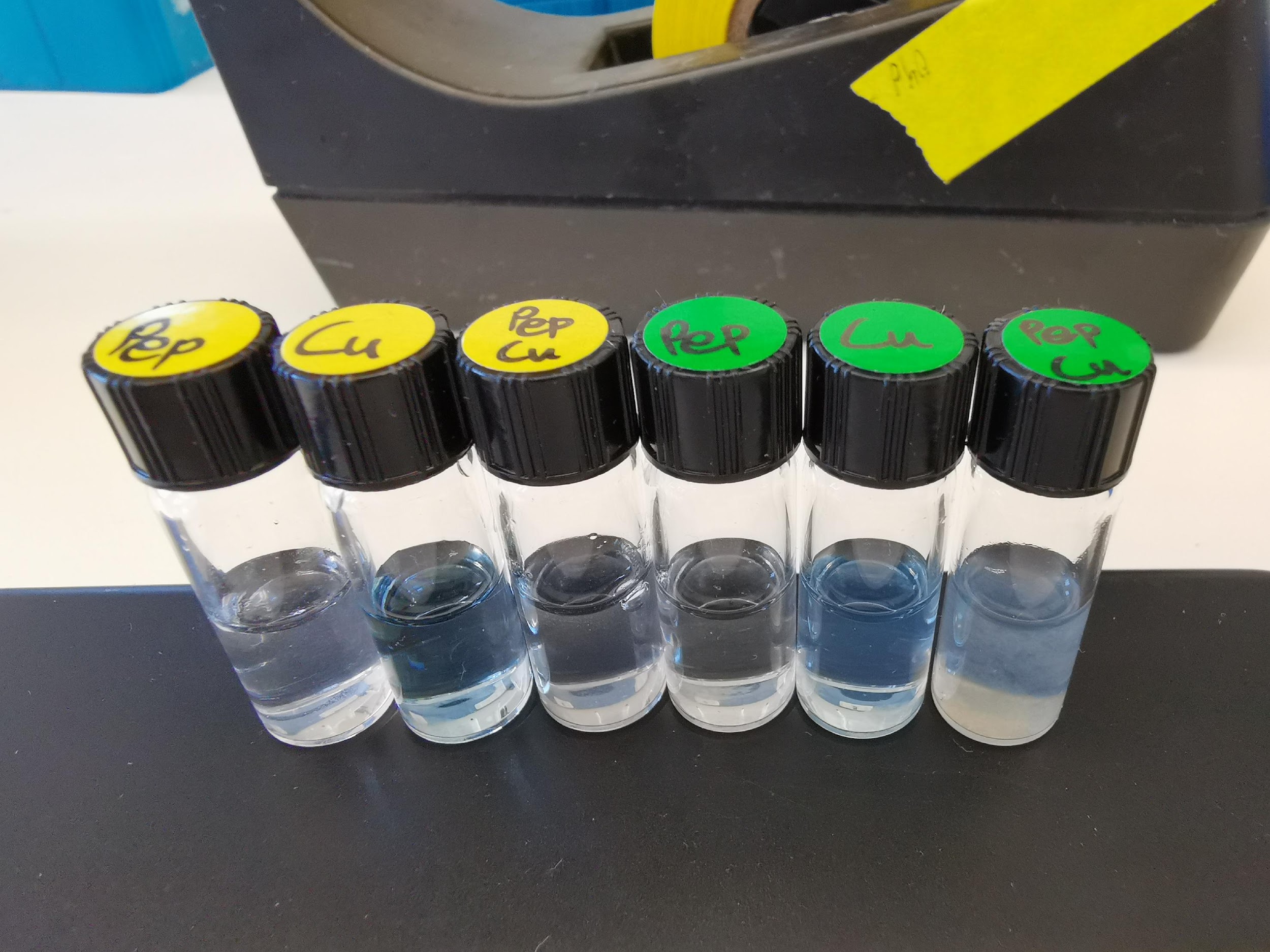


**FIGURE 2 |** A photo of the solutions of the peptide, CuCl_2_, and a mixture of the peptide and CuCl_2_ without NaCl in the buffer (yellow lids) or with NaCl in the buffer (green lids).


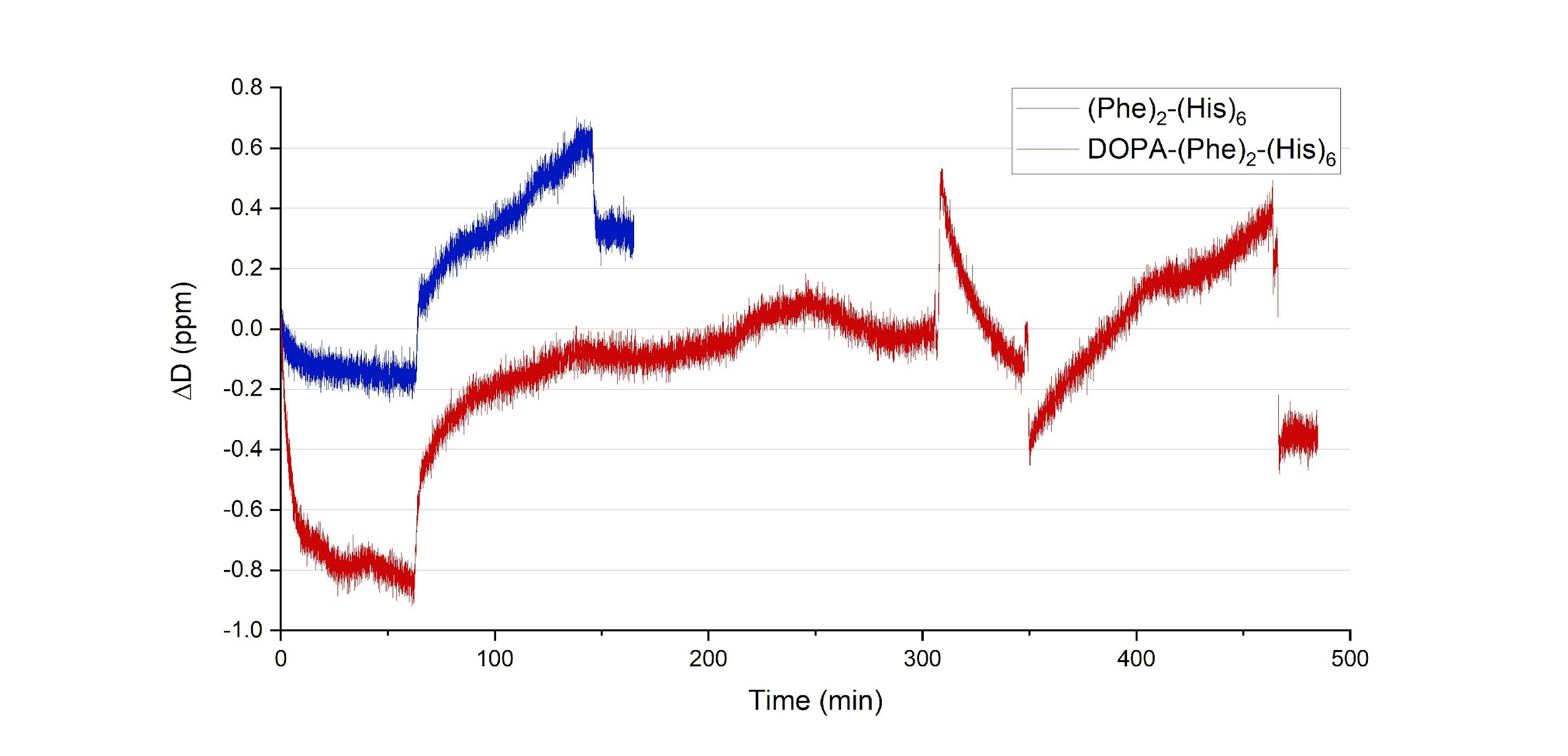


**FIGURE 3 |** The 5^th^ overtones of the dissipation of the peptide (red) and the peptide without DOPA (blue) from the real-time QCM-D measurements.


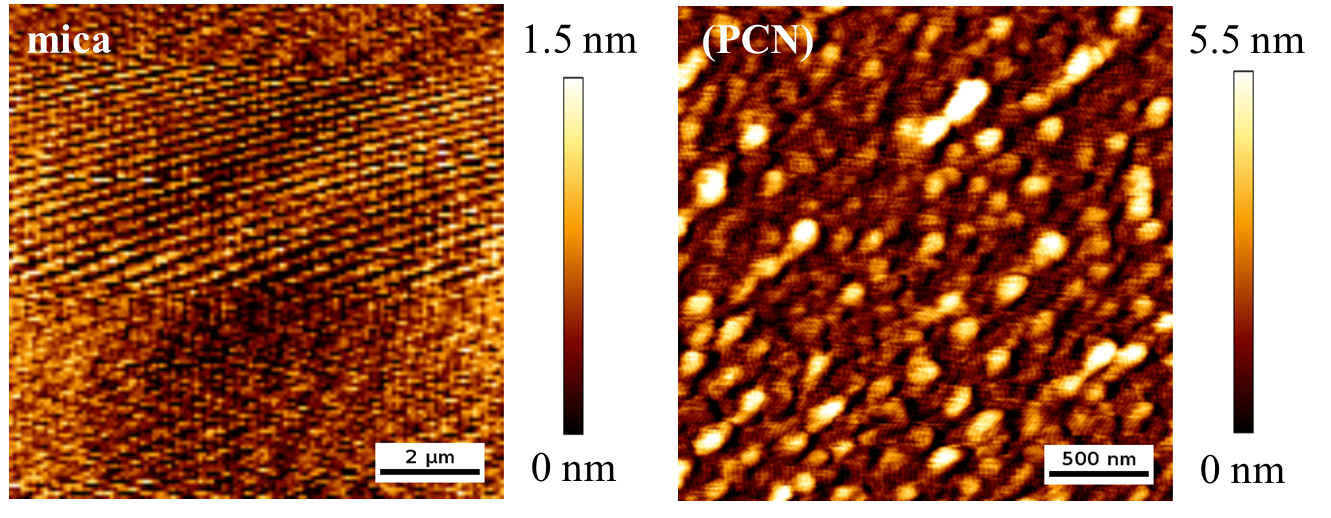


**FIGURE 4 |** AFM topography images of a clean mica surface and a PCN-coated mica surface.


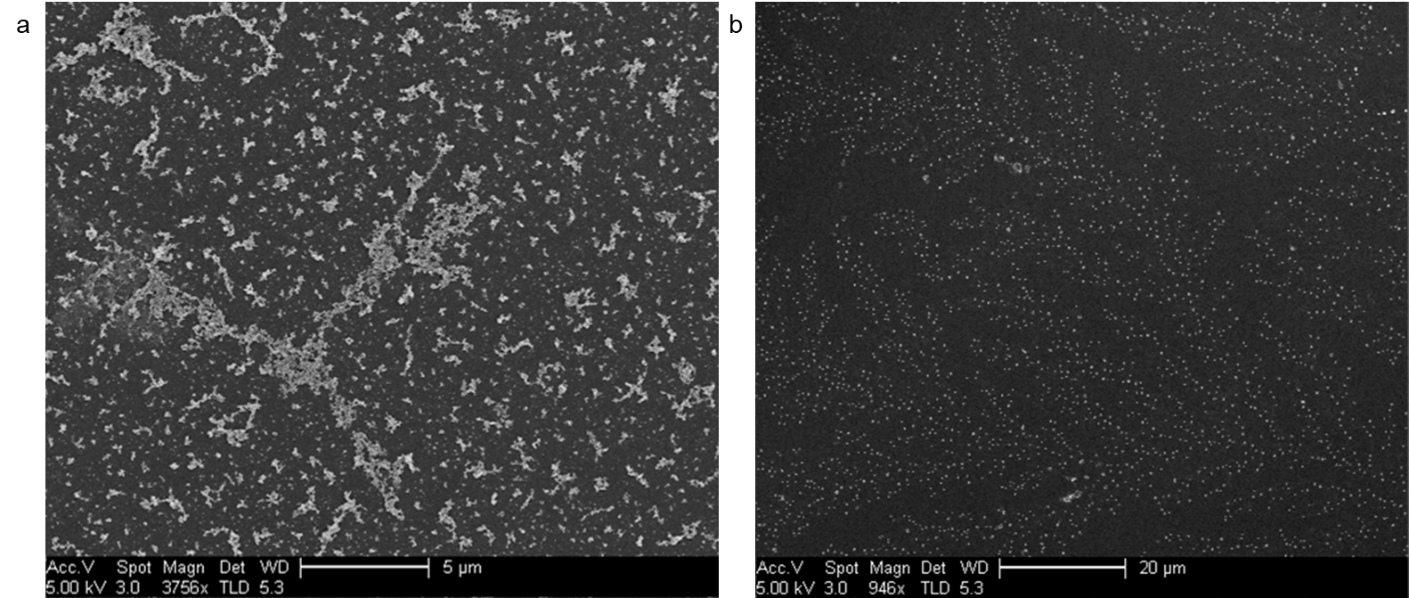


**FIGURE 5 |** (**A**) SEM image of the peptide network-like nanostructures on a PCN-coated surface. (**B**) SEM image of the CuNPs embedded on the peptide nanostructures.


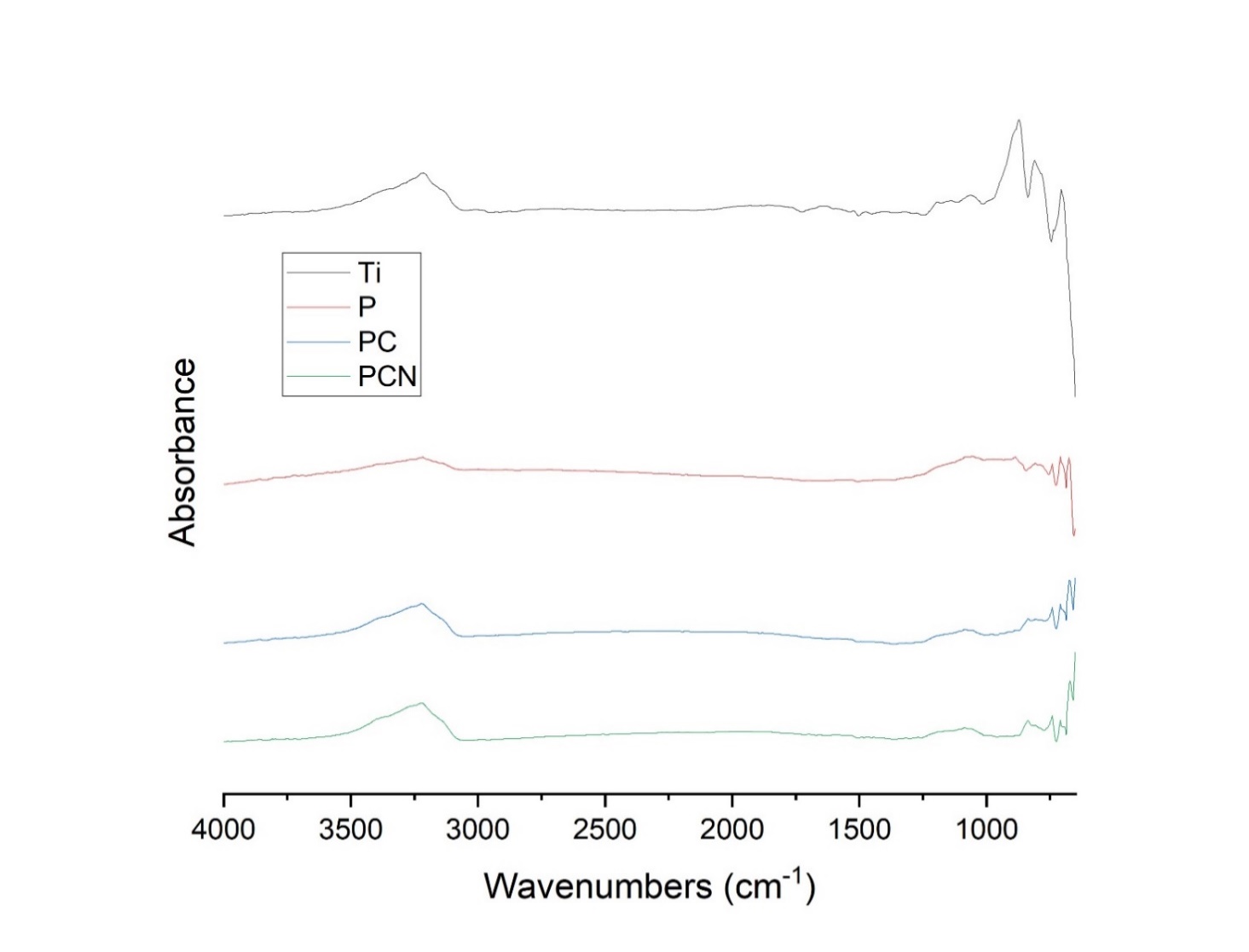


**FIGURE 6 |** ATR-FTIR spectra of bare Ti (black), P-coated Ti surface (red), PC-coated TI surface (blue), and PCN-coated Ti surface (green).


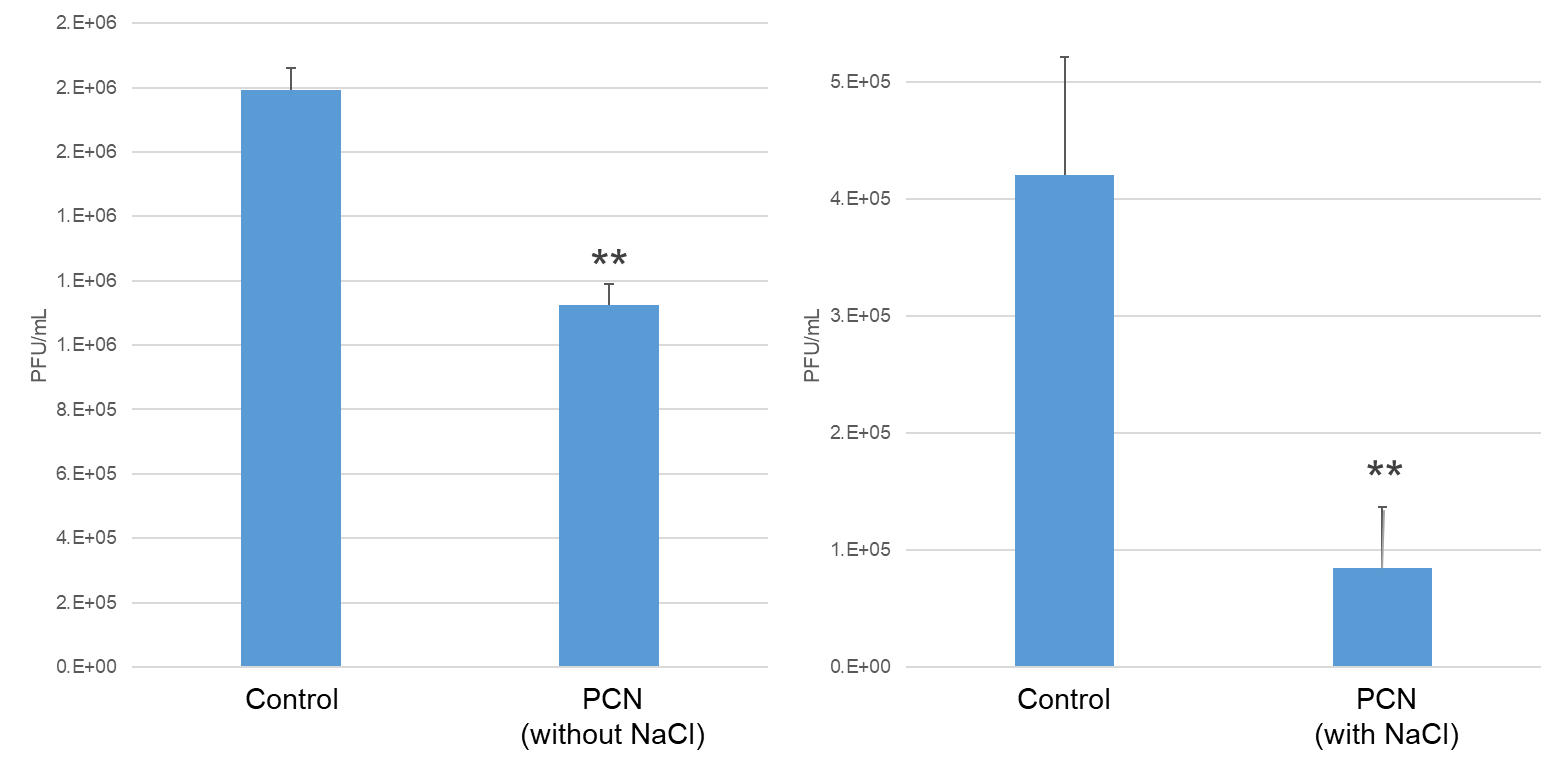


**FIGURE 7 |** Plaque assay results of a clean Ti surface (control) and a PCN-coated surface without NaCl in the buffer (left) or with NaCl in the buffer (right).


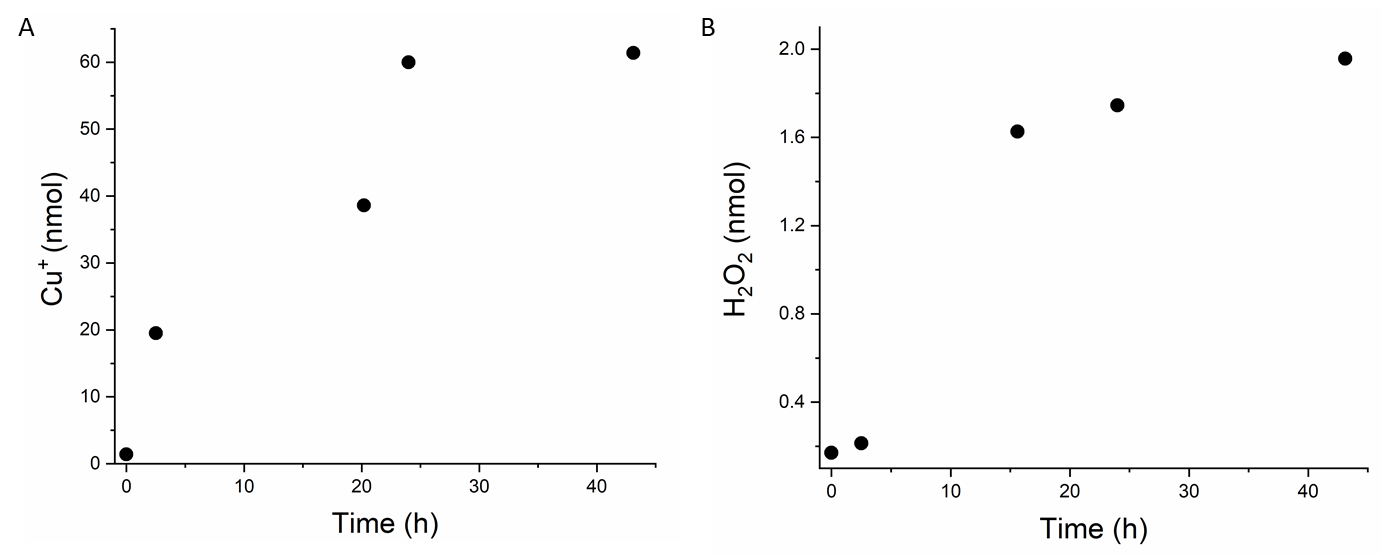


**FIGURE 8 |** (**A**) The release of Cu(I) ions from a PCN-coated surface vs. time, determined using an BCA assay. (**B**) The release of H_2_O_2_ from a PCN-coated surface vs. time, determined using an ABTS assay.

**References**

Barth, A. (2007). Infrared spectroscopy of proteins. *Biochim. Biophys. Acta – Bioenerg.* 1767, 1073–1101. doi:10.1016/j.bbabio.2007.06.004.

Bindig, U., Gersonde, I., Meinke, M., Becker, Y., and Müller, G. (2003). Fibre-optic IR-spectroscopy for biomedical diagnostics. in *Spectroscopy* (IOS Press), 323–344. doi:10.1155/2003/172702.

Haris, P. I., and Chapman, D. (1995). The conformational analysis of peptides using fourier transform IR spectroscopy. *Biopolymers* 37, 251–263. doi:10.1002/bip.360370404.

Maity, S., Nir, S., Zada, T., and Reches, M. (2014). Self-assembly of a tripeptide into a functional coating that resists fouling. *Chem. Commun.* 50, 11154–11157. doi:10.1039/c4cc03578j.

Miller, L. M., Bourassa, M. W., and Smith, R. J. (2013). FTIR spectroscopic imaging of protein aggregation in living cells. *Biochim. Biophys. Acta – Biomembr.* 1828, 2339–2346. doi:10.1016/j.bbamem.2013.01.014.
